# Role of the *ADCY9* gene in cardiac abnormalities of the Rubinstein-Taybi syndrome

**DOI:** 10.1186/s13023-020-01378-9

**Published:** 2020-04-22

**Authors:** Yueheng Wu, Yu Xia, Ping Li, Hui-Qi Qu, Yichuan Liu, Yongchao Yang, Jijin Lin, Meng Zheng, Lifeng Tian, Zhuanbin Wu, Shufang Huang, Xianyu Qin, Xianwu Zhou, Shaoxian Chen, Yanying Liu, Yonghua Wang, Xiaofeng Li, Hanshi Zeng, Hakon Hakonarson, Jian Zhuang

**Affiliations:** 1 Guangdong Cardiovascular Institute, Guangdong Provincial Key Laboratory of South China Structural Heart Disease, Guangdong Provincial People’s Hospital, Guangdong Academy of Medical Sciences, Guangzhou, Guangdong China; 2 Prenatal Diagnosis Center, Department of Obstetrics and Gynecology, Guangdong Provincial People’s Hospital, Guangdong Academy of Medical Sciences, Guangzhou, Guangdong China; 3 Center for Applied Genomics, The Children’s Hospital of Philadelphia, Philadelphia, PA USA; 4 Shanghai Model Organisms Center Inc, Shanghai, China; 5 Department of Pediatrics and Division of Human Genetics, University of Pennsylvania, Philadelphia, PA USA

**Keywords:** *ADCY9*, Congenital heart defects, *MMP9*, Rubinstein-Taybi syndrome

## Abstract

**Background:**

Rubinstein–Taybi syndrome (RTS) is a rare, congenital, plurimalformative, and neurodevelopmental disorder. Previous studies have reported that large deletions contribute to more severe RTS phenotypes than those caused by CREBBP point mutations, suggesting a concurrent pathogenetic role of flanking genes, typical of contiguous gene syndromes, but the detailed genetics are unclear.

**Results:**

This study presented a rare case of Rubinstein-Taybi (RT) syndrome with serious cardiac abnormalities. Based on the clinical and genetic analysis of the patient, the *ADCY9* gene deletion was highlighted as a plausible explanation of cardiac abnormalities. In *adcy9* morphant zebrafish, cardiac malformation was observed. Immunofluorescence study disclosed increased macrophage migration and cardiac apoptosis. RNA sequencing in zebrafish model highlighted the changes of a number of genes, including increased expression of the mmp9 gene which encodes a matrix metalloproteinase with the main function to degrade and remodel extracellular matrix.

**Conclusions:**

In this study, we identified a plausible new candidate gene *ADCY9* of CHD through the clinical and genetic analysis of a rare case of Rubinstein-Taybi (RT) syndrome with serious cardiac abnormalities. By functional study of zebrafish, we demonstrated that deletion of *adcy9* is the causation for the cardiac abnormalities. Cardiac apoptosis and increased expression of the MMP9 gene are involved in the pathogenesis.

## Introduction

Congenital heart defects (CHDs) are the most common congenital malformations with an incidence of 1% of live births in developed countries. These are also the leading cause of birth-defect-related deaths [1]. Some CHDs are also directly associated with ventricular dysfunction, and heart failure (HF) might be more frequent among pediatric patients with these structural abnormalities [2]. To date, 212 genes have been identified of mutations causing CHDs in human [3], which highlights the complexity of the pathogenesis of CHDs. The identification of CHD causal genes is not only helpful to understand the pathogenesis, but also important in clinical management, such as prenatal molecular diagnosis, management of comorbidities, and selection of the most appropriate surgical interventions.

In the current study, we identified a plausible new candidate gene *ADCY9* of CHD through the clinical and genetic analysis of a rare case of Rubinstein-Taybi (RT) syndrome with serious cardiac abnormalities. By functional study of zebrafish, we demonstrated that deletion of *adcy9* is the causation for the cardiac abnormalities.

## Methods

### Clinical case analysis

The clinical analysis was based on a male neonatal case with cardiac abnormalities admitted to our hospital. The parents and the older sister of the case had normal phenotype. His mother was a 34-year-old female, G4P1Ab2, who had the history of twice induced abortions, once cesarean section, and received obstetric examinations on irregular basis. At 23 weeks of gestation, there was no obvious fetal abnormality shown by the pelvic B-ultrasound results with 3.0 MHz probe. Gestational diabetes of the mother was found at 26 weeks of gestation, with the OGTT results of @fasting 4.71 mmol/L; @1 h 8.82 mmol/L; @2 h 6.84 mmol/L. Through diet control, blood glucose of the mother was well controlled. At 27 weeks of gestation, pelvic B-ultrasound results revealed fetal heart malformation. The imaging showed that the ascending aorta and aortic arch of the fetus were thin, the aorta was narrowed, and the oval hole was large. Fetal umbilical cord blood was collected at 27 gestational weeks, and fetal chromosome, Hb electrophoresis, perinatal infection tests (including cytomegalovirus, herpes simplex virus, rubella virus, and Toxoplasma gondii), and chromosomal microarray (CMA) examination were performed on umbilical cord blood. Chromosome exam of fetal umbilical cord blood showed 46, XN, 21pst polymorphism, and the CMA result showed 16p13.3 microdeletion (chr16: 3,721,533-4,242,948; hg19). The perinatal infection tests were negative. The parents refused to take the CMA examination. A clinical diagnosis of RT syndrome with cardiac abnormalities was made, and the parents chose to continue the pregnancy after informed consent.

Caesarean section was performed at 38 + 4 gestational weeks. The infant was delivered with a weight of 3460 g with Apgar score of 10/10 points. Broad thumbs/toes and short fingers of the infant were observed, while distal joints of the two thumbs broadened more significantly. Muscle strength of the limbs was normal, and no other obvious deformity was found in the outward appearance. On the third day after birth, cardiac ultrasound showed patent ductus arteriosus (PDA), aortic valve dysplastic tricuspid with stenosis, aortic arch dysplasia, minor secondary atrial septal defect, moderate tricuspid regurgitation, severe pulmonary hypertension, and cardiac hypertrophy (asymmetric right ventricular hypertrophy). Annulus diameter of aortic valve cannot be determined (Z value = − 3.8), with aorta 7.5 mm, diameter of aortic arch 4.2 mm(Z value = − 4), isthmus diameter 4.9 mm(Z value = − 1), distal diameter 8.2 mm (Z value = 1.8), arterial catheter 4.4 mm, and bidirectional shunt (online only materials). With the clinical diagnosis of RT syndrome with cardiac abnormalities confirmed, the vital signs and respiratory and circulation status of the patient were closely monitored after birth.

On the third day after birth, the patient developed shortness of breath, increased heart rate and hematochezia. Physical examination showed decreased blood pressure and decreased oxygen saturation, abdominal distension and edema of lower limbs and scrotum. Blood tests revealed decreased platelets and hemoglobin, with carbon dioxide retention. Necrotizing enterocolitis and respiratory failure were diagnosed consequently. Mechanical ventilation, fasting, gastrointestinal decompression, infusion of red blood cells and platelets were administered, and anti-inflammatory treatment was enhanced. However, the general condition of the patient was continuously deteriorating, and the patient left our hospital with his parents at 2 weeks after birth. The final diagnosis at our hospital was: 1 Rubinstein-Taybi syndrome (RTS); 2 congenital heart disease, aortic arch dysplasia, patent ductus arteriosus, atrial septal defect, severe pulmonary hypertension; 3 necrotizing enterocolitis; 4 respiratory failure.

### Zebrafish model

#### Zebrafish maintenance

Adult wild-type AB strain zebrafish were maintained at 28.5 °C on a 14 h light/10 h dark cycle [4]. Five to six pairs of zebrafish were set up for nature mating each time. On average, 200 ~ 300 embryos were generated. Embryos were maintained at 28.5 °C in fish water (0.2% Instant Ocean Salt in deionized water). The embryos were washed and staged according to standard procedures [5]. The establishment and characterization of the *TG (zlyz: EGFP)* transgenic lines has been described elsewhere [6]. The zebrafish facility for this study at Shanghai Research Center for Model Organisms is accredited by the Association for Assessment and Accreditation of Laboratory Animal Care (AAALAC) International.

#### Zebrafish microinjections

The morpholino (MO) study was designed by Gene Tools, LLC (http://www.gene-tools.com/). Antisense MO was microinjected into fertilized one-cell stage embryos according to standard protocols [7]. The sequence of the *adcy9* exon 3-intron 3 splice-blocking morpholino (*adcy9*-e3i3-MO) was 5′- TAGTTTGGTTCATCATGTACCTTGC − 3′, and the sequence for the standard control morpholino was 5′- CCTCTTACCTCAGTTACAATTTATA − 3′ (Gene Tools). For the *adcy9* gene knock-down experiment, 4 ng of control-MO or *adcy9*-e3i3-MO was used per injection. Total RNA was extracted from 30 to 50 embryos per group in TriPure Isolation Reagent (Roche) according to the manufacturer’s instructions. RNA was reverse transcribed using the PrimeScript RT reagent Kit with gDNA Eraser (Takara). Primers spanning *adcy9* exon 2 (forward primer: 5‘- TGGGCTCTCAGTGTGGATGTT -3’) and exon 4 (reverse primer: 5‘- GGAAGGATTGCCGAGTGGT -3’) were used for the RT-PCR analysis for confirmation of the efficacy of the *adcy9*-e3i3-MO (Supplementary Figure 1). The sequences of the primer *ef1α* used as the internal control were 5‘- GGAAATTCGAGACCAGCAAATAC -3’ (forward) and 5‘- GATACCAGCCTCAAACTCACC -3’ (reverse).

### Acridine orange staining for apoptosis

Embryos injected with Control-MO and *adcy9*-e3i3-MO were immersed in 5 μg/ml Acridine orange (AO, acridinium chloride hemi-[zinc chloride], Sigma-Aldrich) in fish water for 60 min at 3 d postfertilization (dpf). Next, zebrafish were rinsed thoroughly in fish water for three times (5 min/wash) and anaesthetized with 0.016% MS-222 (tricaine methanesulfonate, Sigma-Aldrich, St. Louis, MO). Zebrafish were then oriented on their lateral side and mounted with 3% methylcellulose in a depression slide for observation by fluorescence microscopy.

### In vivo macrophage migration assays

To evaluate macrophage migration in zebrafish, fertilized one-cell embryos of *TG (zlyz: EGFP)* transgenic lines were injected with 4 ng *adcy9*-e3i3-MO or control-MO. After treatment, all embryos were incubated at 28.5 °C. At 6 dpf, embryos were anesthetized with 0.016% MS-222 (tricaine methanesulfonate, Sigma-Aldrich, St. Louis, MO) and the number of macrophages recruited to the heart was counted.

### Quantitative real-time PCR

Total RNA was extracted from 30 to 50 embryos per group in Trizol (Roche) according to the manufacturer’s instructions. RNA was reverse transcribed using the the PrimeScript RT reagent Kit with gDNA Eraser (Takara). Quantification of gene expression was performed in triplicates using Bio-rad iQ SYBR Green Supermix (Bio-rad) with detection on the Realplex system (Eppendorf). Relative gene expression quantification was based on the comparative threshold cycle method (2 − ΔΔCt) using *ef1α* as endogenous control gene. Primer sequences are given in Supplementary Table 1.

### Image analysis

Embryos and larvae were analyzed with Nikon SMZ 1500 Fluorescence microscope and subsequently photographed with digital cameras (5.0 MP). A subset of images was adjusted for levels, brightness, contrast, hue and saturation with Adobe Photoshop 7.0 software (Adobe, San Jose, California) to optimally visualize the expression patterns. Quantitative image analyses were processed using image based morphometric analysis (NIS-Elements D3.1, Japan) and ImageJ software (U.S. National Institutes of Health, Bethesda, MD, USA; http://rsbweb.nih.gov/ij/). Inverted fluorescent images were used for processing. Positive signals were defined by particle number using ImageJ. Ten animals for each treatment were quantified and the total signal per animal was averaged.

### RNA sequencing

In order to better understand the mechanisms of *adcy9*, we performed RNA sequencing in zebrafish model. Control-MO injected embryos and embryos injected with *adcy9*-e3i3-MO were frozen at 3 dpf (1 dpi) for RNA-seq analysis. Two biological replicates of 30 embryos each assay were analyzed in each experimental group. RNA was purified using RNAqueous Total RNA isolation kit (Thermo Fisher). Library preparation and next-generation sequencing were performed at the CCHMC Core Facility using Illumina HiSeq 2500 (75 read length, single sided, 20 M reads per sample). Quality check of the RNA-SEQ reads was performed using Fastqc (http://www.bioinformatics.babraham.ac.uk/projects/fastqc). Reads with low quality adapter content and over-represented sequences were trimmed using SeqPrep (https://github.com/jstjohn/SeqPrep)and Sickle (https://github.com/najoshi/sickle). We mapped and quantified the trimmed RNA-SEQ reads using HISAT2(https://ccb.jhu.edu/software/hisat2/index.shtml) to latest Zebrafish genome assembly GRCz11.92 for each sample at default thresholds.

Differential expression tests of RNA-seq data were performed using the Cuffdiff package in Cufflink2.2.1 [8], and the GTF gene template file was based on *Danio rerio* version GRCz11. Library sizes (i.e. sequencing depths) are normalized by the classic-fpkm method. The normality of data distribution was rechecked by visual confirmation. Each protein coding gene was compared the estimated expression level based on measurement of Fragments Per Kilobase of transcript per Million (FPKM). The genes were considered as differentially expressed only if their adjusted *p* value (q value) less or equal than 0.05, and the gene enrichment analysis in Gene Ontology (GO, Data Release 2019–01) and biological pathways were done by DAVID v6.8 Bioinformatics platform [9]. A pathway was considered significant by Bonferroni corrected *p* < 0.05.

### Statistical analysis

All data are presented as mean ± SEM. Statistical analysis and graphical representation of the data were performed using GraphPad Prism 5.0 (GraphPad Software, San Diego, CA). Statistical significance was tested using a Student’s t test or χ^2^ test as appropriate. Statistical significance is indicated by *, where *P* < 0.05, and ***, where *P* < 0.0001.

## Results

### Clinical genetic analysis

The clinical phenotype of the child includes: 1 broad thumbs/toes; 2 patent foramen ovale, ASD and PDA; 3 aortic development abnormalities (i.e. aortic annulus, ascending aortic coarctation); 4 cardiac hypertrophy; 5. heart failure; 6 respiratory failure; 7 small intestinal necrosis.

There are several special phenomena worthy of attention in the patient’s medical history. First, the ultrasound exam showed normal aortic development at 23 weeks of gestation, but showed that the ascending aorta and aortic arch were slightly thinner at 27-week, while the aortic annulus and aortic arch were severely constricted after birth. This change indicates that aortic dysplasia in the current case occurred in the late aortic development and deteriorated over time. Secondly, the patient’s heart development is basically normal, in which case the aortic malformation is generally surgically treatable. However, after the child was born, he developed rapidly rare complications, including ventricular hypertrophy and heart failure, respiratory failure, and small intestinal necrosis. These suggest a highly complicated nature of the patient’s disease, but not a simple cardiac malformation. Thirdly, that the patient’s left ventricular development was normal until the child was born. In general, early aortic dysplasia in the fetus (e.g. abnormal aortic dysplasia can be detected by ultrasound at as early as 16 weeks) can lead to left ventricular dysplasia, mainly due to insufficient blood supply [10], but which did not occur in this case. The relatively small effect on left ventricular development in this case might be due to late and less serious aortic dysplasia occurred in the third trimester. Overall, this is a rare and special RTS case, whereas serious complications occurred after birth. Because of these, we decided to investigate its underlying molecular mechanisms through state-of-art technologies and approaches.

### Zebrafish model

The CMA analysis of the patient showed the deletion region of 3,721,533-4,242,948 [hg19] at 16p13.3 and three genes, *TRAP1*, *CREBBP,* and *ADCY9,* are mapped to this region. Mutations, deletions, and duplications of the *CREBBP* gene cause RTS, which is typically characterized by broad thumbs and toes [11]. In animal models, no cardiac abnormalities were recorded [12]. As shown in the literature, large *CREBBP* deletions contribute to more severe RTS phenotypes than point mutations, implying a concurrent pathogenetic role of flanking genes, a phenomenon typically known as contiguous gene syndromes [13, 14]. Therefore, it is reasonable to hypothesize that in addition to the *CREBBP* gene, other genes in the same deleted region may be involved in the disease of the current patient. For the other two genes *TRAP1* and *ADCY9*, the function of *TRAP1* has been relatively clear [15, 16]. *TRAP1* encodes an intra-mitochondrial protein, highly homolog to members of the Hsp90 family, which play fundamental roles in protein folding, protein degradation and signal transduction [17]. Despite these, the *Trap1* knockout mice did not have heart abnormalities [18]. Therefore, we proposed *ADCY9* as a plausible candidate to explain the severe heart abnormalities observed in the current patient.

The *ADCY9* gene is conserved across multiple species. Compared with zebrafish, the protein and DNA similarity rates of human *ADCY9* are 65.2 and 64.7% respectively. To exam the function of *ADCY9* in heart abnormalities, we designed an *adcy9* knockdown experiment in zebrafish via morpholino-modified antisense oligonucleotides. RT-PCR and Sanger sequencing confirmed that the *adcy9* MO experiment was successful, and the target sequence was inserted into the intron 3 of the *adcy9* gene in the MO group.

**Fig. 1:**
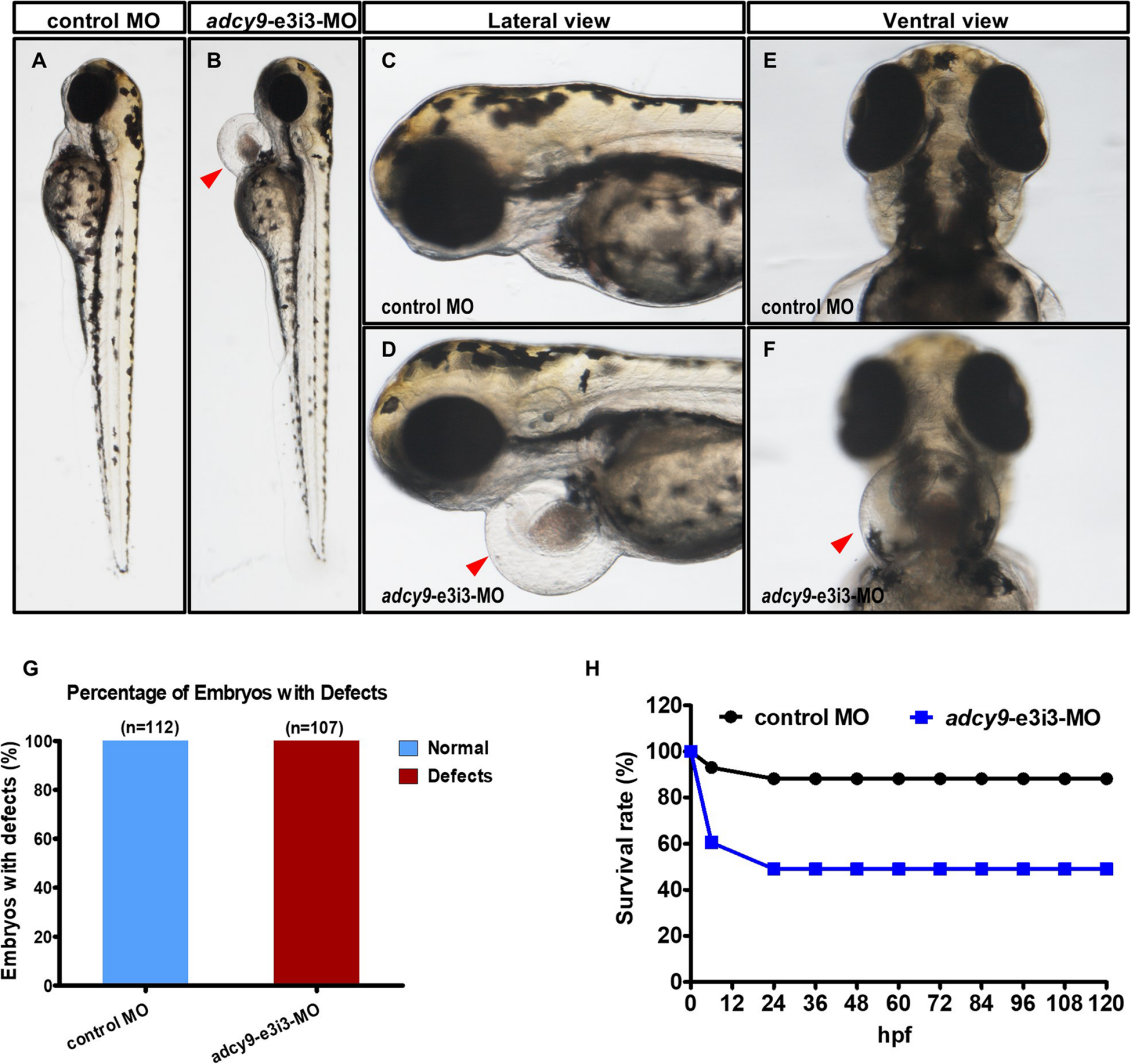
Phenotypes of *adcy9* zebrafish morphants. (**a**-**f**) Gross morphology at 3-dpf. Compared with control MO, knock down *adcy9* present pericardial oedema (**b**, **d**, **f**, red arrow) (Heart beat is visible in the control fish, but is abnormal in *adcy9*-e3i3-MO injected fish (Supplementary Movie 1, Supplementary Movie 2). The bar graph in Panel G shows the percentage of embryos with development defects. **h** A time-course plot of percent survival in control vs. *adcy9* morphants for 5 days


Heart beat is visible and normal in the control zebrafish, but is abnormal in *adcy9*-e3i3-MO injected zebrafish (Fig. 1, Supplementary Movie 1, Supplementary Movie 2). Compared with the control zebrafish, the zebrafish with the *adcy9* knockdown has slower heart rate, pericardial edema, small ventricles, cardiac hypertrophy, and atrial blood stagnation. All embryos (100%) had development defects. The *adcy9* morphant zebrafish has a high mortality rate of 50% at birth. The cardiac malformation of the *adcy9* morphant zebrafish is mainly presented in the following two aspects: 1. the ventricle of the *adcy9* morphant zebrafish is smaller than that of the control zebrafish, indicating ventricular dysplasia in the zebrafish; 2. the *adcy9* morphant zebrafish generally have symptoms such as decreased heart rate, pericardial edema, cardiac hypertrophy, and atrial congestion. These indicate that the *adcy9* morphant zebrafish have severe acute heart failure.

**Additional file 4 Supplementary Movie 1**. Ventral view.



**Additional file 5 Supplementary Movie 2**. Lateral view.


#### Immunofluorescence study on macrophage and apoptosis

**Fig. 2:**
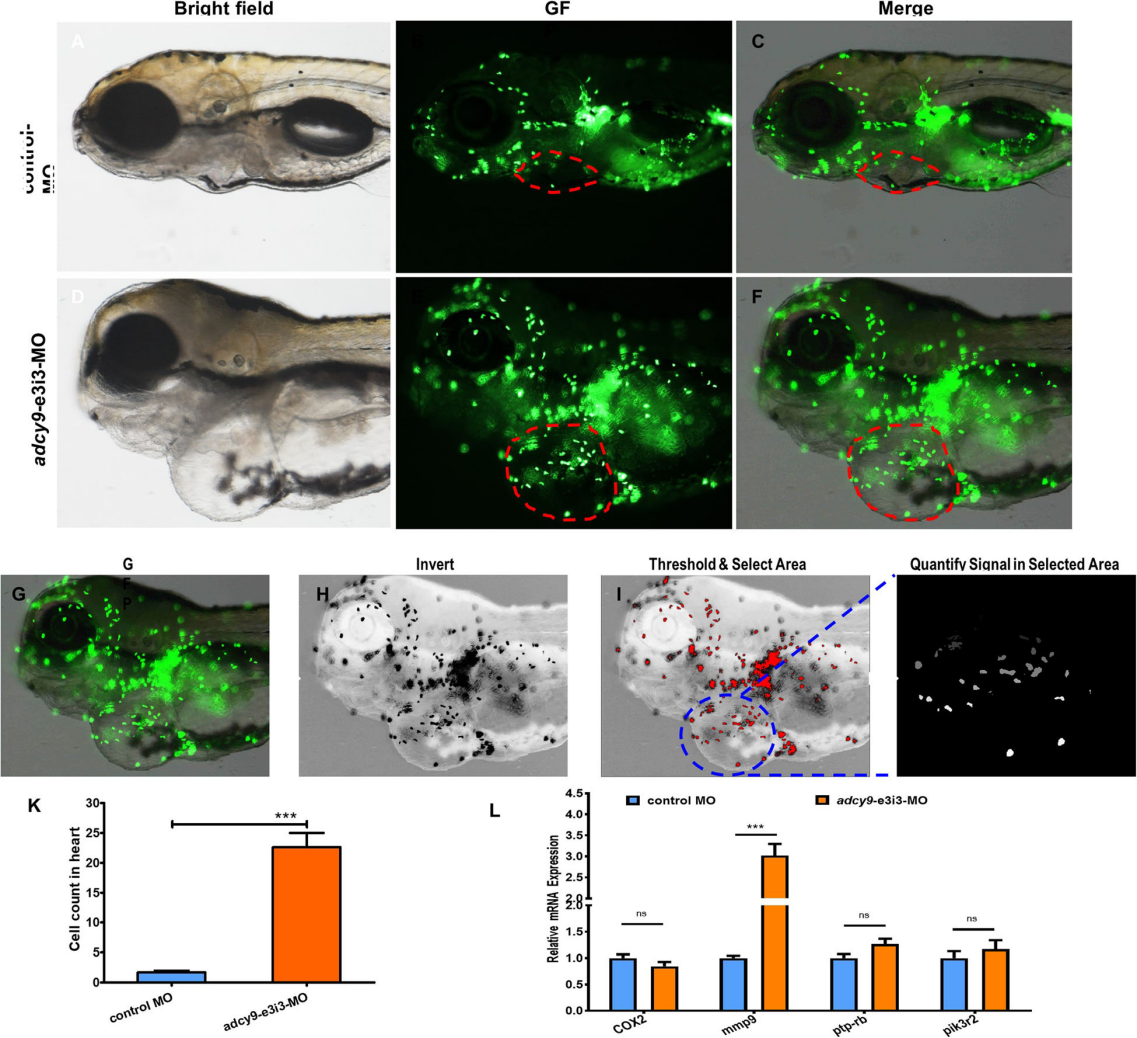
Effects of *adcy9* knock down on macrophage migration. **a**-**f** Representative bright field and fluorescent images of *TG (zlyz: EGFP)* larvae at 6 days postfertilization (dpf). Control MO injected fish show the normal distribution of labeled cells (**b** and **c**). Compared with control fish, embryos injected with *adcy9*-e3i3-MO present potent macrophage migration in heart (**e** and **f**, red circle area). **g**-**k** Quantification of the macrophage number at heart shows a 13.3-fold increased in *adcy9* morphants. Columns, mean; bars, SEM (*n* = 10; Student’s t test; ***P < 0.0001;). dpf, days post fertilization. **l** Endogenous efnb2a, COX2, mmp9, ptp-rb and pik3r2 in control and adcy9 morphants assessed by qRT-PCR (*n* = 6–10 individual embryos). ns, not significant

**Fig. 3:**
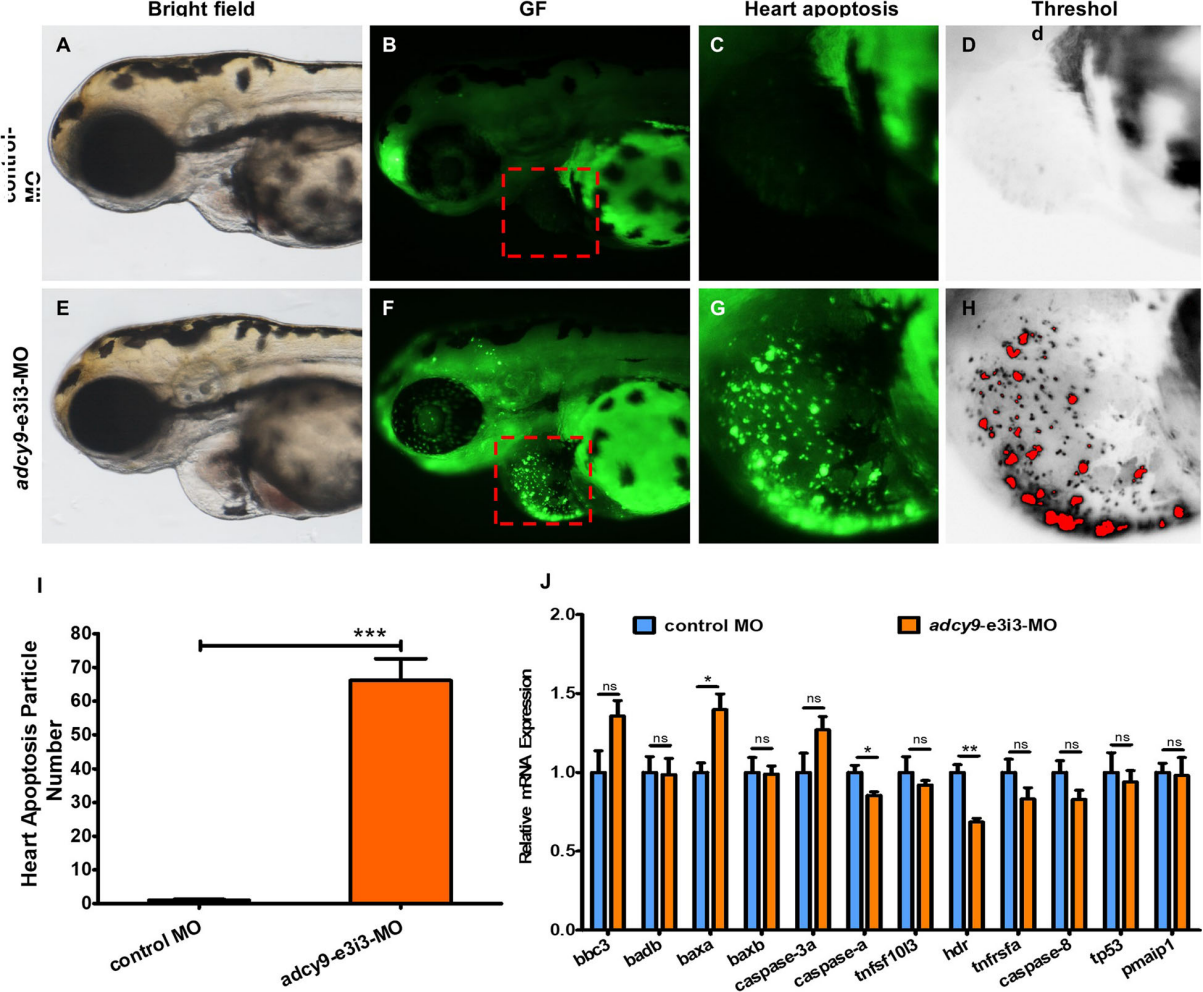
Morpholino knock down of *adcy9* induces potent apoptosis in heart. Control-MO injected embryos and embryos injected with *adcy9*-e3i3-MO were stained with acridine orange (AO) at 3-dpf. Heart apoptotic cells are visible as bright green spots or red spots (**f**-**h**), and less bright homogenous green staining is unspecific background staining. **a**-**d** Control-MO injected zebrafish exhibited few or no apoptotic cells in heart. In contrast, significantly increased staining was observed throughout the heart in zebrafish injected with 4 ng *adcy9*-e3i3-MO (F-H). The red boxed regions are shown at higher magnification in the right panels. **i** Quantification of apoptosis particle number at heart shows a 66.1-fold increased in *adcy9* morphants. Error bars, s.e.m.; ***P < 0.0001(*n* = 10; Student’s t test;); **a**-**h**: lateral view, anterior, left. Dpf, days post fertilization. **j** Endogenous *tnfsf10l3*, *hdr*, *tnfrsfa*, *caspase-8*, *tp53*, *pmaip1*, *bbc3*, *badb*, *baxa*, *baxb*, *caspase-3a* and *caspase-a* in control and *adcy9* morphants assessed by qRT-PCR (n = 6–10 individual embryos). ns, not significant

The effect of *adcy9* knockdown on macrophage migration was observed by immunofluorescence technique (Fig. 2). Compared with control fish, embryos injected with adcy9-e3i3-MO showed increased macrophage migration, including the heart. The effect of *adcy9* knockdown on zebrafish apoptosis was also observed by immunofluorescence technique. In contrast to control fish, apoptosis was specifically observed in embryonic hearts of *adcy9* knockdown zebrafish (Fig. 3). Real-time PCR confirmed that the apoptotic indicator in zebrafish, baxa, had increased expression, while caspases and HDR had reduced expression.

#### RNA-seq results

To investigate the molecular mechanisms of acute heart failure and high mortality of *adcy9* MO zebrafish, we performed RNA sequencing analysis. The RNA-seq results of differential gene expression analysis comparing zebrafish with or without *adcy9* knockdown are shown in Supplementary Table 2. Most significantly, *ela2*(encoding elastase 2) and *cyp1a* (Human ortholog gene *CYP1A1*, encoding a member of the cytochrome P450 superfamily of enzymes) had decreased expression in the *adcy9* morphant zebrafish. In contrast, these genes had increased expression:

*mmp9* (Human ortholog gene *MMP9*, encoding matrix metallopeptidase 9, involved in the breakdown of extracellular matrix in embryonic development);

*syngap1a* (Human ortholog gene *SYNGAP1*, encoding synaptic Ras GTPase activating protein 1);

*fosl1a* (Human ortholog gene *FOSL1*, encoding FOS like 1, AP-1 transcription factor subunit, regulating cell proliferation, differentiation, and transformation);

*kank2* (Human ortholog gene *KANK2*, encoding KN motif and ankyrin repeat domains 2, involved in cytoskeletal formation by regulating actin polymerization);

*acp5a* (Human ortholog gene *ACP5*, encoding acid phosphatase 5, tartrate resistant);

*pxnb* (Human ortholog gene *PXN*, encoding paxillin, a cytoskeletal protein).

Limited by the sample size of the RNA-seq study, we got 17 genes with q-value< 0.10, which means 10% of these genes (~ 2 genes) are false positive. Considering the critical roles of *mmp9* in developmental growth and regeneration, we did a realtime PCR experiment and confirmed its higher expression in the morphant zebrafish.

**Fig. 4:**
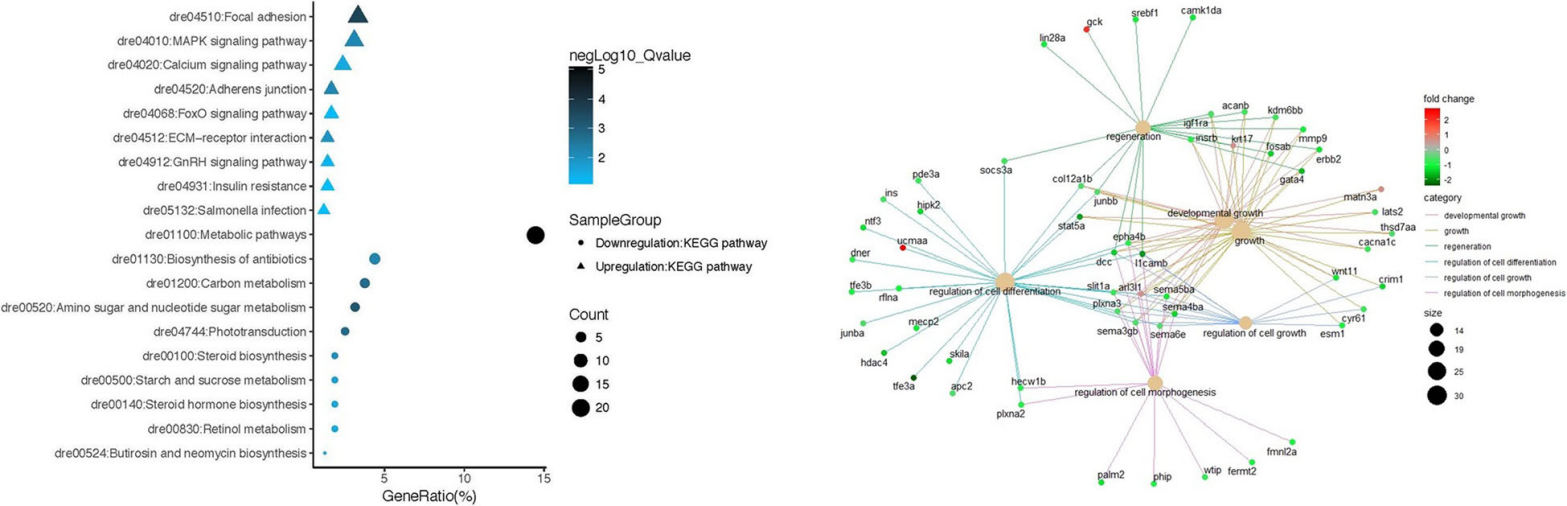
Scatter plot for KEGG enrichment results. The Gene Ratio is the ratio of differentially expressed gene numbers annotated in this pathway term to all gene numbers annotated in this pathway term. The greater the Gene Ratio, the greater the degree of pathway enrichment. A negLog10_Q value is the corrected *p* value ranging from 1 to 10, and a higher value indicates greater pathway enrichment

Consequently, we performed GO and Kyoto Encyclopedia of Genes and Genomes (KEGG) analyses on up-regulated and down-regulated genes (Fig. 4). The GO Biological Processes (BP), the GO Molecular Functions (MF), and the KEGG pathways, highlighted in this study, are shown in Supplementary Table 3. A complete list from the pathway analysis are shown in Supplementary Table 4.

## Discussion

### ADCY9: a plausible new candidate gene of CHD

The most common causes of RTS are de novo microdeletions at 16p13.3, as in this case because the parents have normal phenotype [19]. About 30% of this syndrome has also cardiac malformations, e.g. patent foramen ovale, ASD, and patent ductus arteriosus [20–22]. These heart malformations are usually not serious, and the patients can generally live to adulthood [11, 22]. In contrast, in the current case the phenotype of the patient is more serious and complicated. In addition to the broad thumbs and toes, there were patent foramen ovale, atrial septal defect (ASD), patent ductus arteriosus, aortic developmental defects (aortic annulus, ascending aorta coarctation). Severe phenotypes that are not common in RT syndrome were also seen in this case, including cardiac hypertrophy, left ventricular dysplasia syndrome, heart failure, respiratory failure, and small intestinal necrosis [23, 24].

*ADCY9* encodes adenylate cyclase type 9, an enzyme producing the ubiquitous second messenger cAMP [25]. The *ADCY9* gene is composed of five exons and is highly conserved across different species. The previous study by Jin et al. has identified two heterozygous and loss-of-function mutations (LOF, including one splicing mutation and one frame-shifting mutation) in the *ADCY9* gene in a large collection of CHD patients, but no *ADCY9* mutation in controls [3]. Studies have identified the association between single nucleotide polymorphisms (SNP) of *ADCY9* (rs1967309) and body mass index (BMI) [26, 27]. Studies showed also that *ADCY9* polymorphism is associated with cardiovascular disease [28] and cardiovascular outcomes in dyslipidemia treatment [29], but controversies remain [30–32]. Based on the literature and the genetic tests of the current patient, we speculate that the deletion of *ADCY9* gene may be associated with severe heart disease in patients. As shown in our study, *adcy9* morphant zebrafish have very high mortality and deformity rates, indicating the important function of *adcy9* in the general and cardiac development of zebrafish.

### Myocardial apoptosis in adcy9 morphant zebrafish

Cardiac hypertrophy was observed in both the patient and *adcy9* morphant zebrafish. Considering the role of myocardial apoptosis in cardiac hypertrophy, we did fluorescence experiments in zebrafish’s myocardium and confirmed that myocardial apoptosis occurred in the heart of *adcy9* morphant zebrafish. The signaling pathways of apoptosis in both zebrafish and human can be divided into exogenous and endogenous, while both ultimately lead to high expression of baxa [33, 34]. Our results showed high expression of baxa, which confirmed the occurrence of apoptosis in *adcy9* morphant zebrafish. However, the signaling molecules, caspase-a and HDR, of exogenous apoptotic signaling pathways were significantly reduced, suggesting that the apoptosis of *adcy9* morphant zebrafish may come from endogenous pathways, instead of exogenous pathways.

### mmp9 in cardiac abnormalities in adcy9 morphant zebrafish

A number of genes were highlighted in the RNA-seq results to explain the phenotypes of *adcy9* MO zebrafish. Among these genes, *mmp9* has been demonstrated of critical roles in cardiac development. *mmp9* encodes a matrix metalloproteinase whose main function is to degrade and remodel extracellular matrix, and plays critical roles in the formation and function of the placenta and the fetal organs [35]. The *mmp9* protein is mainly produced by macrophages [36]. Our results showed that macrophage migration in MO zebrafish was increased, which is concordant with the high expression of *mmp9*. *mmp9* has been found to be associated with numerous pathological processes, including cancer [37], viral infection [38], and blood lipoprotein levels [39]. In addition, *mmp9* is associated with a variety of cardiovascular diseases, e.g. idiopathic atrial fibrillation [40], aortic aneurysms [41] And heart failure [42, 43]. Toba et al. showed that transgenic overexpression of MMP-9 in macrophages exacerbates the effects of MMP-9 in cardiac aging, leading to greater inflammation and fibrosis, which may exacerbate myocyte hypertrophy [44]. MMP-9 contributes to cardiovascular remodeling, and *mmp9* deletion attenuated thoracic aortic aneurysm formation in mouse model [45]. On the other hand, recent study showed that MMP-9 deficiency augmented angiotensin II-induced abdominal aortic aneurysms [46], related to impair collagen organization and angiogenesis, smooth muscle cell migration and geometrical arterial remodeling [47]. Mmp9 is also associated heart failure [48]. Mmp9 deletion may attenuate age-related myocardial fibrosis and diastolic dysfunction [49]. Circulating MMP-9 has also been shown to be an effective marker of heart failture and dilated cardiomyopathy [42, 50]. Therefore, higher expression of *mmp9* in *adcy9* morphant zebrafish observed in our study may be involved the impairment of extracellular matrix, and cause cardiac abnormalities in zebrafish. There is no known interaction between *ADCY9* and *MMP9*. The overexpression of mmp9 due to adcy9 knock-down is likely a downstream event with a number of genes in-between.

In conclusion, the case with the 16p13.3 microdeletion syndrome reported in this study had serious aortic dysplasia and heart failure. The *ADCY9* gene in the deleted region is a plausible candidate to explain the cardiac abnormalities. The animal study in *adcy9* morphant zebrafish demonstrated that *adcy9* deletion causes cardiac abnormalities. Increased macrophage migration, cardiac apoptosis, and elevated expression of the *mmp9* gene are involved in the pathogenesis. From the clinical aspect, pregnancies with fetal *ADCY9* deletion should be followed closely for aortic dysplasia, even if an initial echocardiogram is normal, to ensure timely surgical correction.

A limitation of this study is that we used the CMA for the genetic testing of the patient. To date, whole exome sequencing (WES) and whole genome sequencing (WGS) have been increasingly used in diagnosing genetic diseases. Compared with WES and WGS, CMA is a well established method, but is relatively old and cannot define the deletion boundary as precise as the former two methods, especially WGS. The zebrafish is a particularly useful model to study gene function in heart development as the embryos can survive without a functional cardiovascular system and blood circulation, by passive diffusion of oxygen [51]. However, we admit that it has limitations. The zebrafish heart has only one atrium and one ventricle, with a primitive outflow tract and without a right ventricle. Therefore, the abnormalities in the patient wouldn’t be fully recapitulated by this zebrafish model.

## Conclusions

In this study, we identified a plausible new candidate gene *ADCY9* of CHD through the clinical and genetic analysis of a rare case of Rubinstein-Taybi (RT) syndrome with serious cardiac abnormalities. By functional study of zebrafish, we demonstrated that deletion of adcy9 is the causation for the cardiac abnormalities. Cardiac apoptosis and increased expression of the MMP9 gene are involved in the pathogenesis.

## Supplementary information

**Additional file 1.** Online only materials

**Additional file 2 Supplementary Figure 1**. Effectiveness of *adcy9* knockdown was confirmed by RT-PCR and sanger sequencing.

**Additional file 3 Supplementary Table 1.** qRT-PCR primers for Zebrafish

**Additional file 6 Supplementary Table 2.** Differential gene expression analysis comparing zebrafish with or without adcy9 knockdown

**Additional file 7.** Supplementary Table 3

**Additional file 8.** Supplementary Table 4

## Data Availability

All data are fully available without restriction.

## Abbreviations

RTS: Rubinstein-Taybi syndrome
RT: Rubinstein-Taybi
CHDs: Congenital heart defects
CHD: Congenital heart disease
CMA: Chromosomal microarray
MO: Morpholino
PDA: Patent ductus arteriosus
KEGG: Kyoto Encyclopedia of Genes and Genomes
GO: Gene Ontology
BMI: Body mass index
ASD: Atrial septal defect
SNP: Single nucleotide polymorphisms
