# Supplementary material for: Role of the ADCY9 gene in cardiac abnormalities of the Rubinstein-Taybi syndrome
Source: Orphanet J Rare Dis. 2020 Apr 22;15:101. doi: 10.1186/s13023-020-01378-9 (PMC7178576; doi:10.1186/s13023-020-01378-9)

## Slide 1
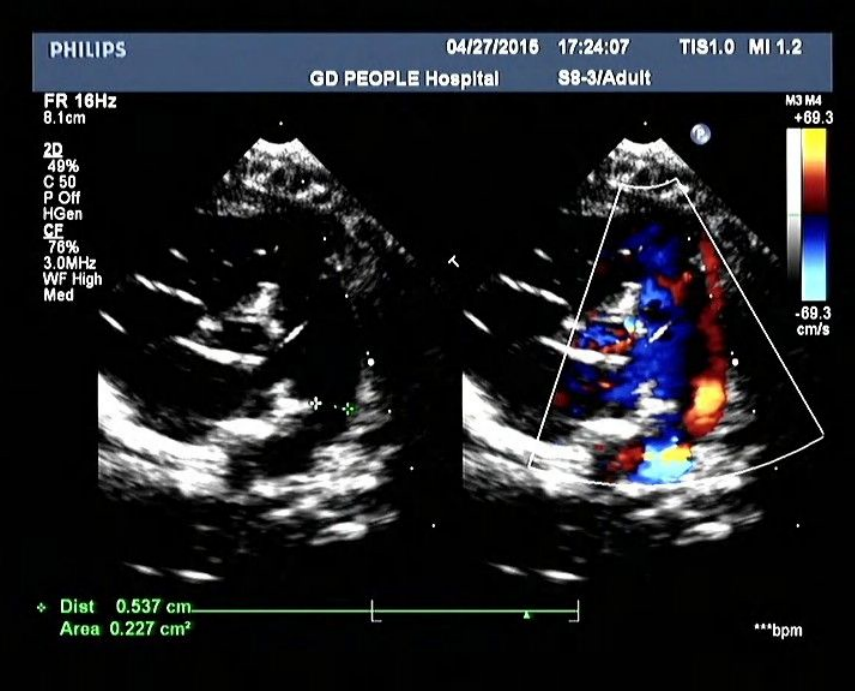

## Slide 2
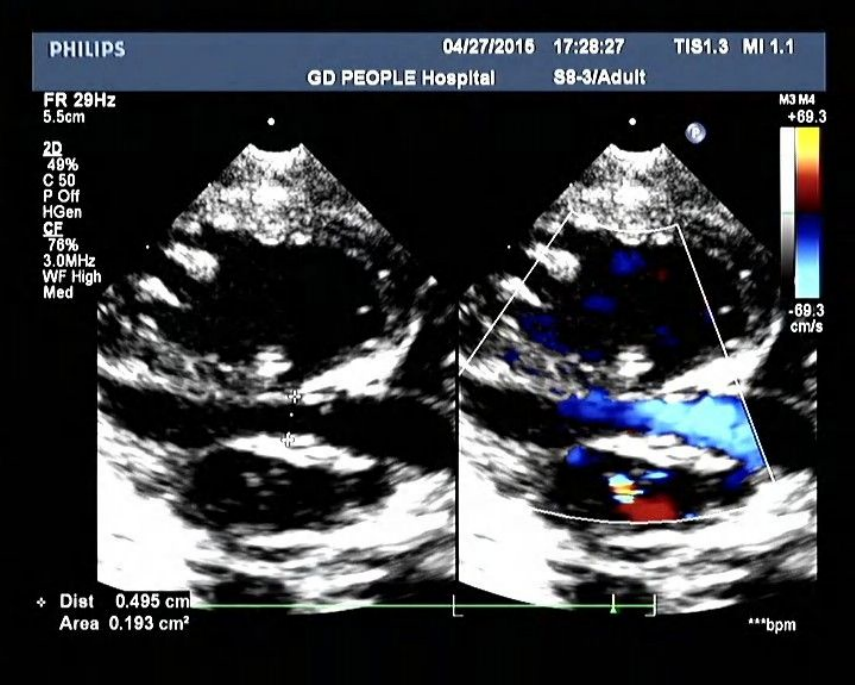

## Slide 3
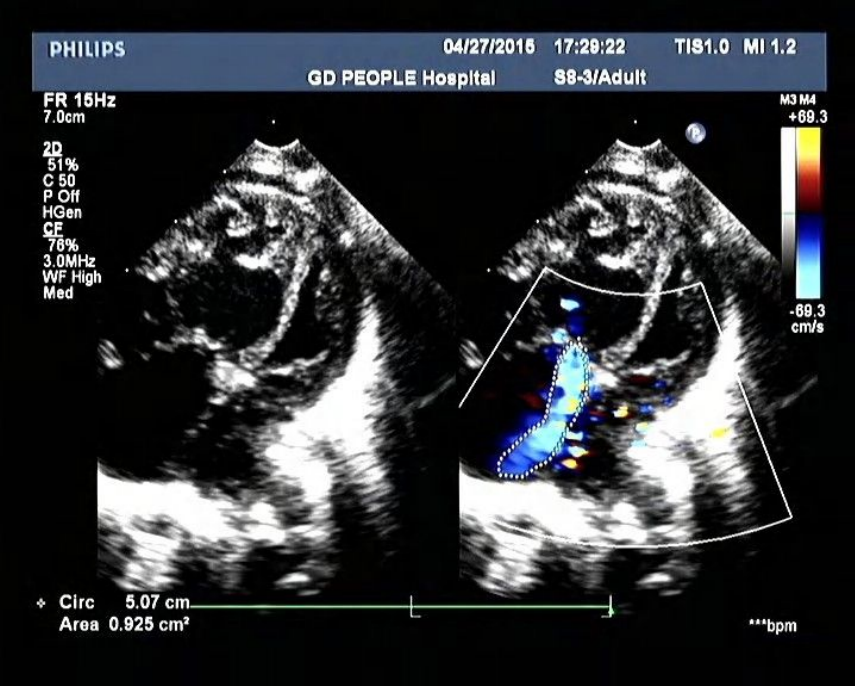

## Slide 4
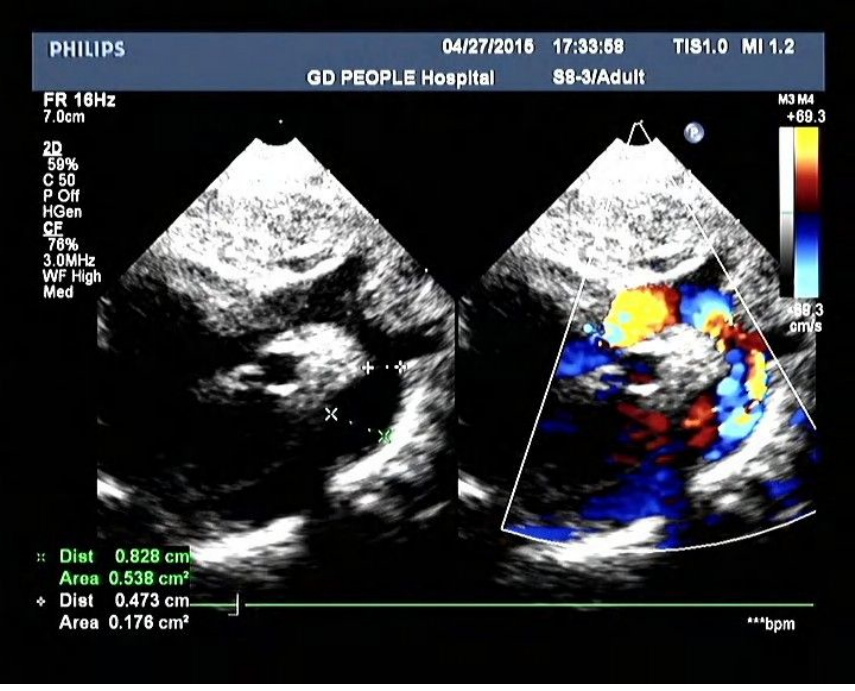

## Slide 5
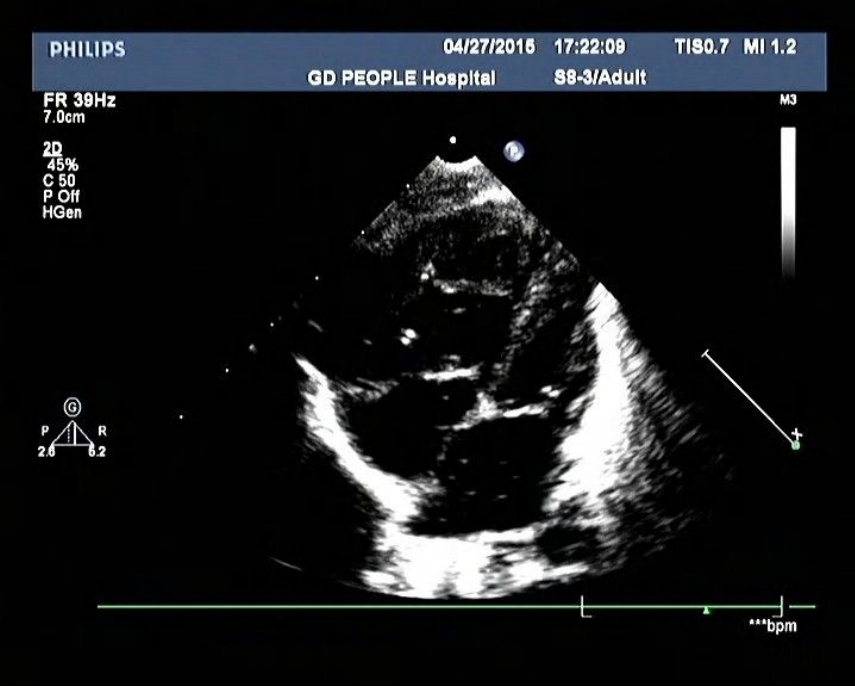

## Slide 6
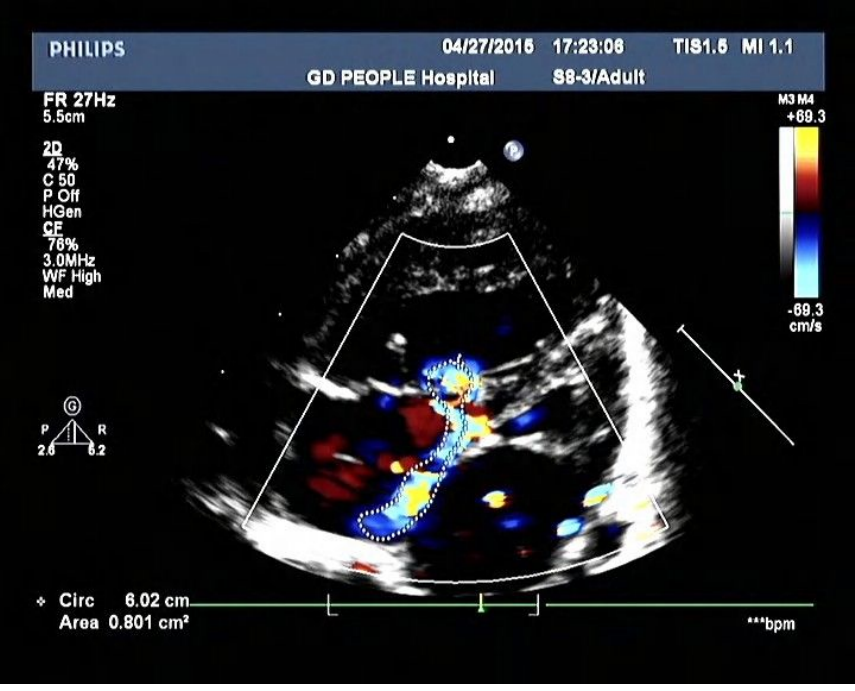

## Slide 7
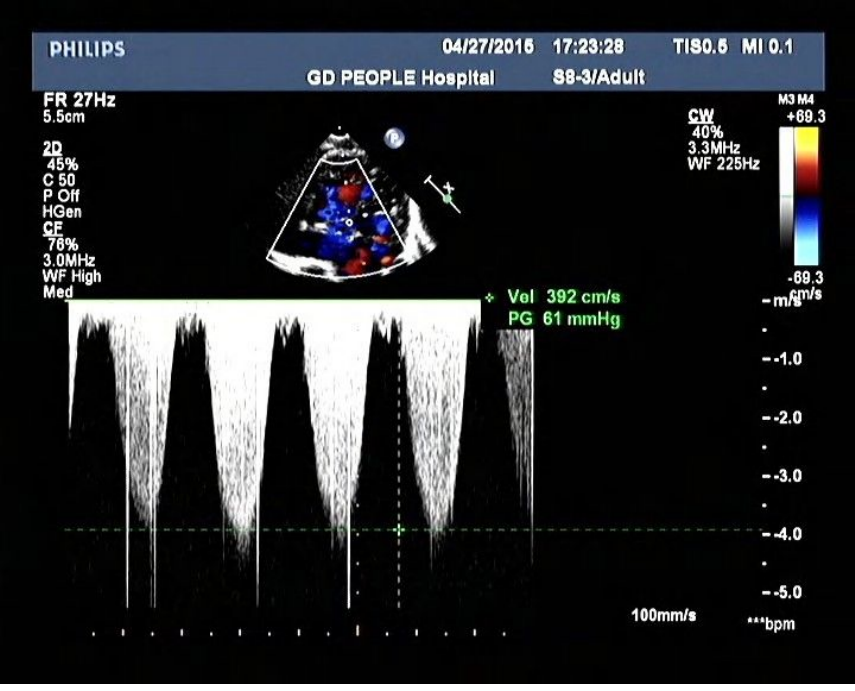

## Slide 8
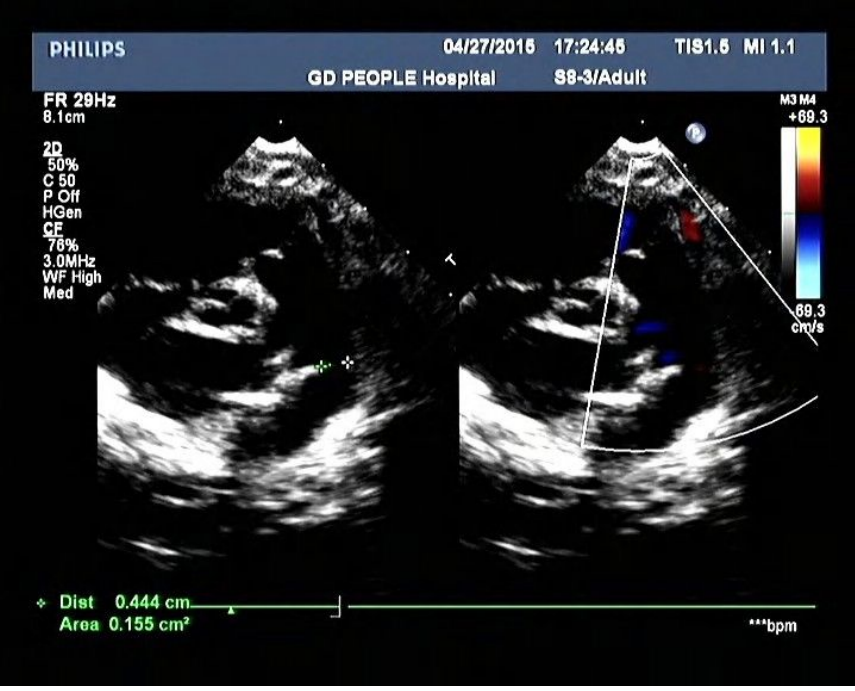

## Slide 9
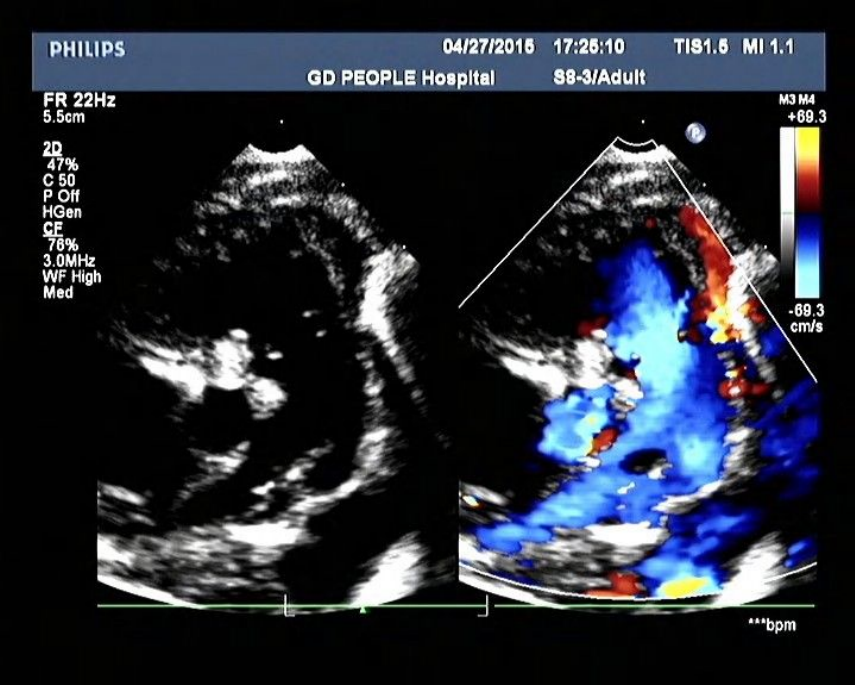

## Slide 10
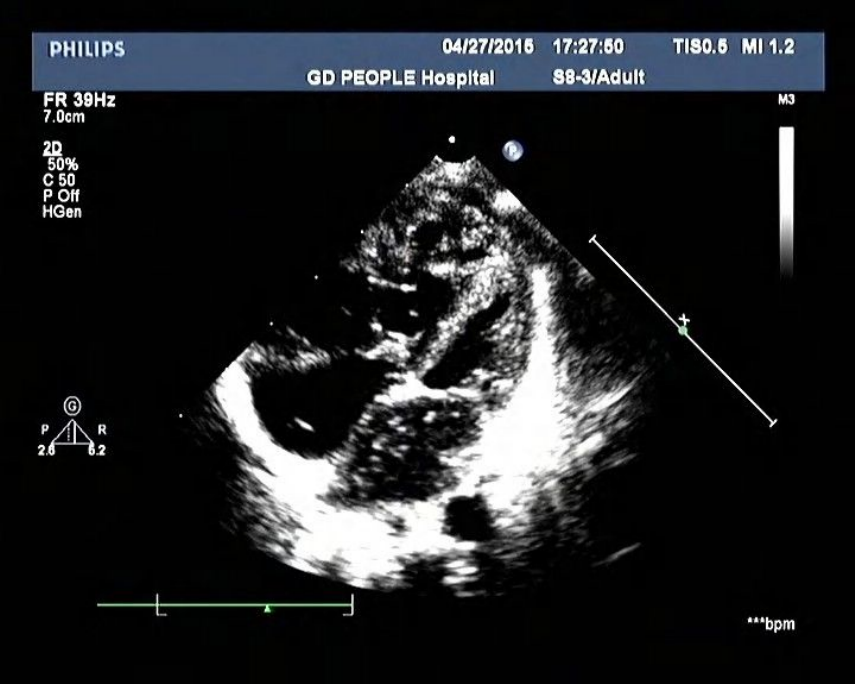

## Slide 11
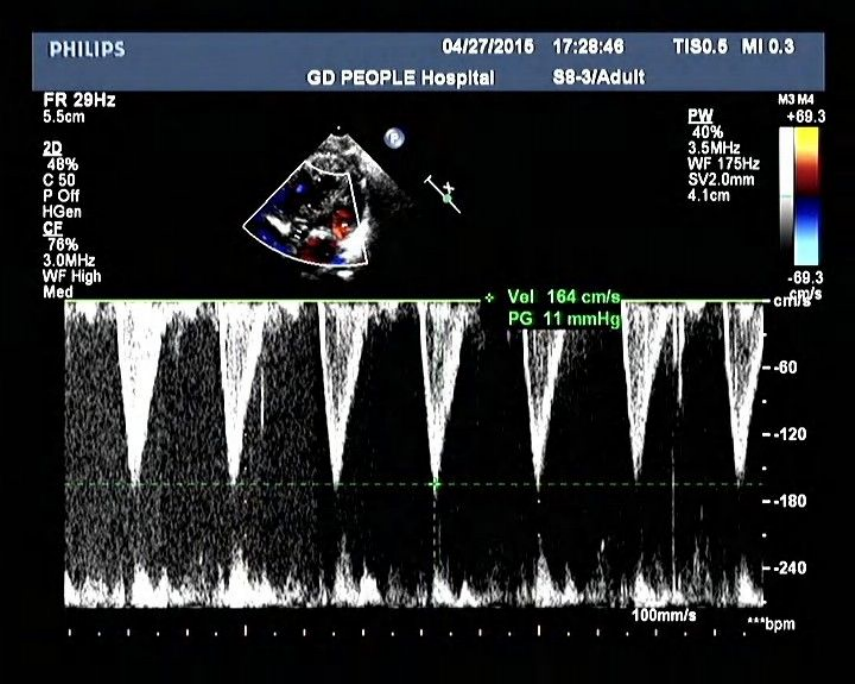

## Slide 12
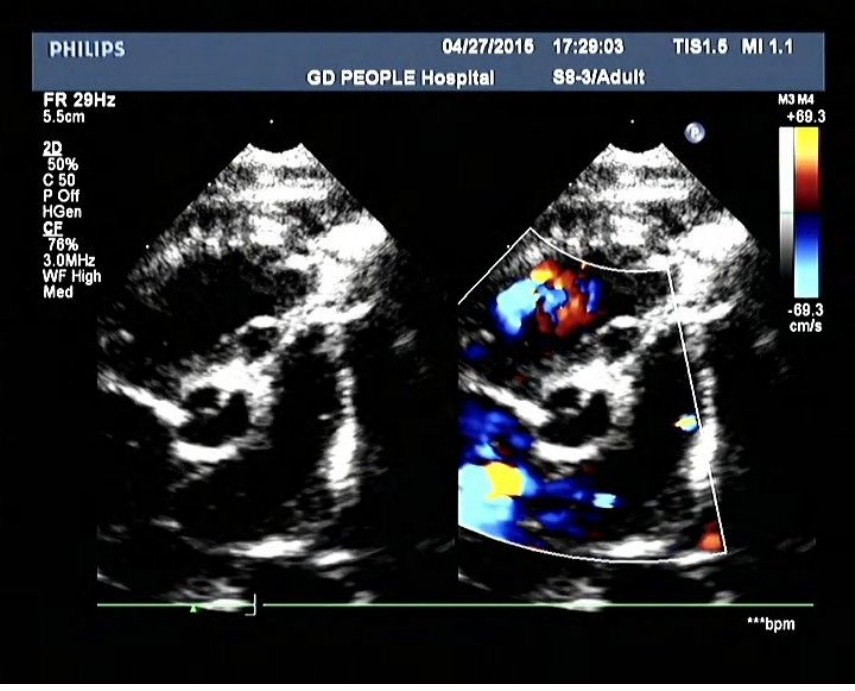

## Slide 13
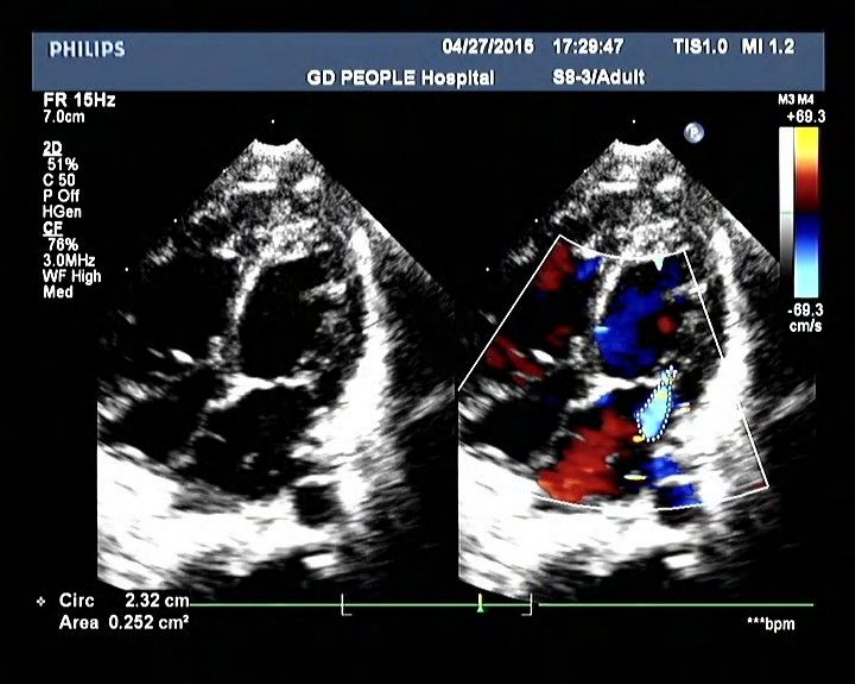

## Slide 14
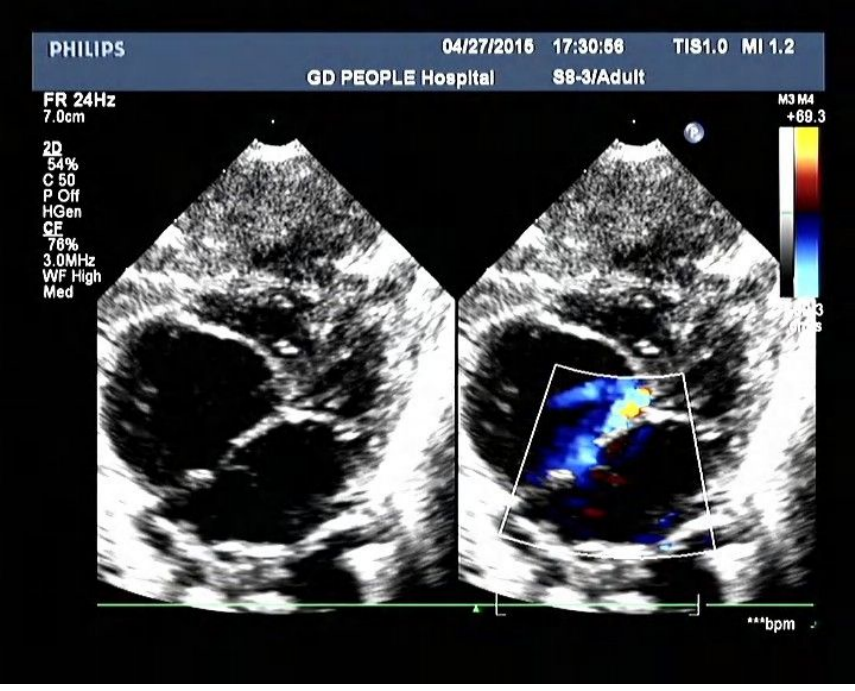

## Slide 15
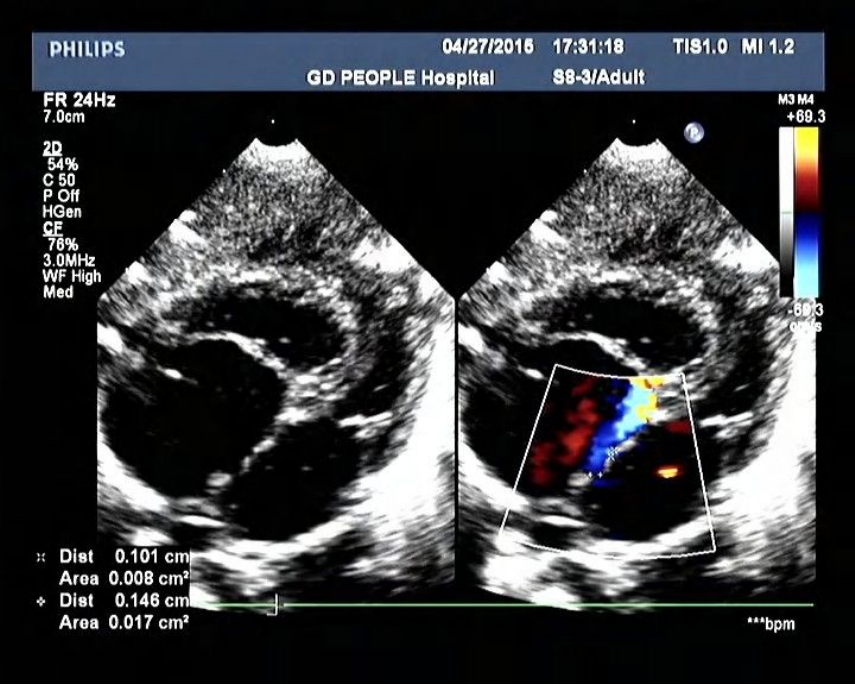

## Slide 16
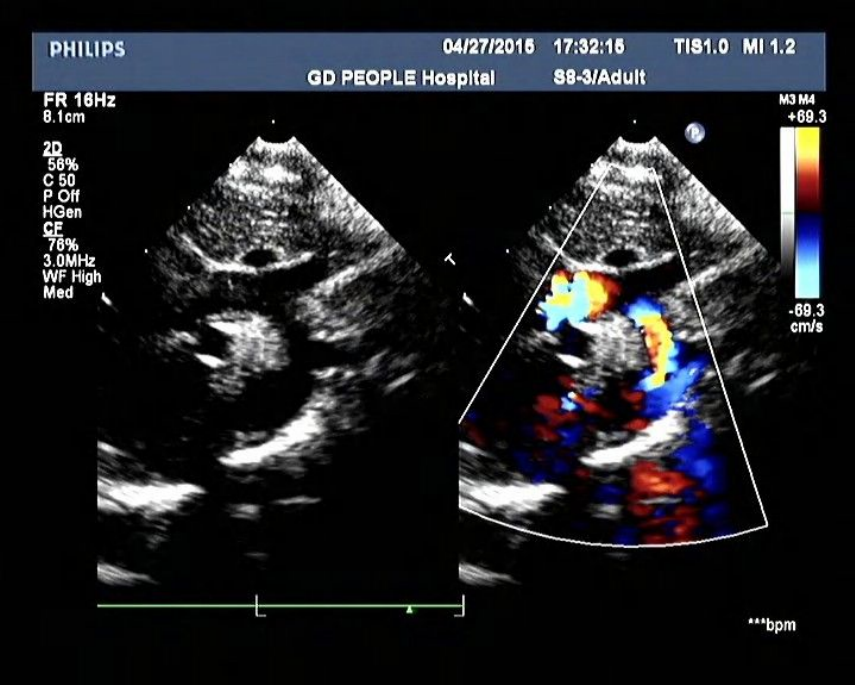

## Slide 17
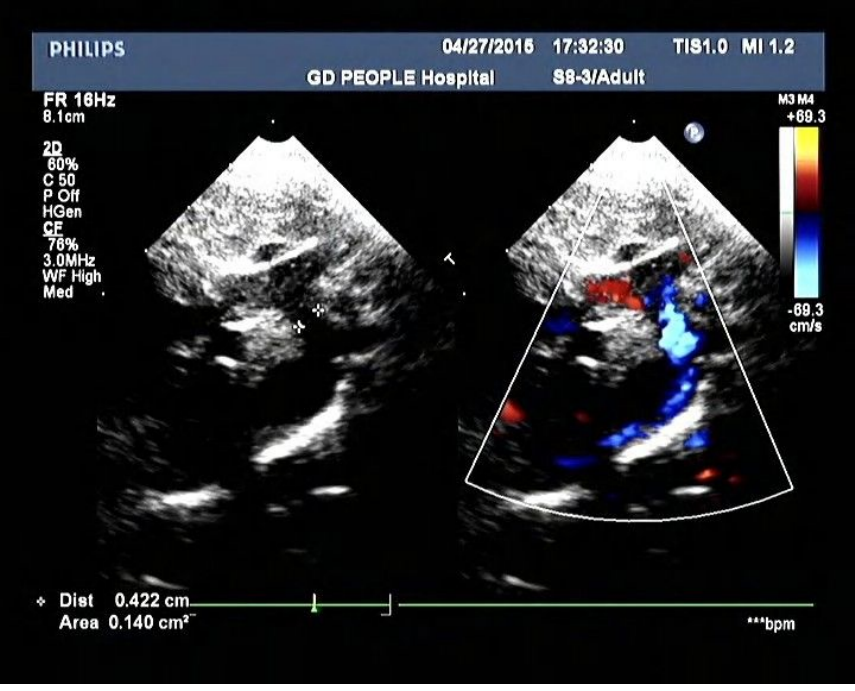

## Slide 18
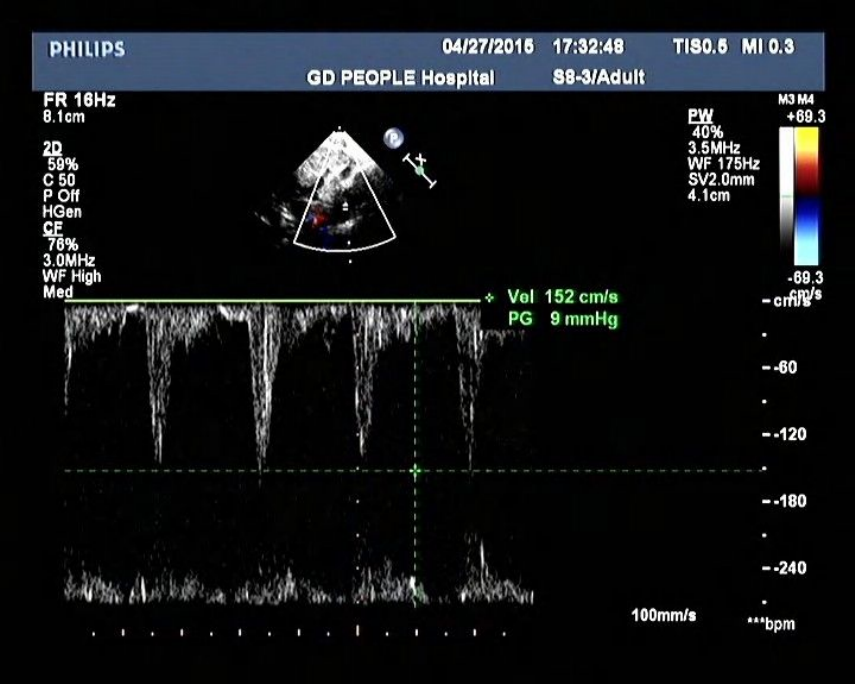

## Slide 19
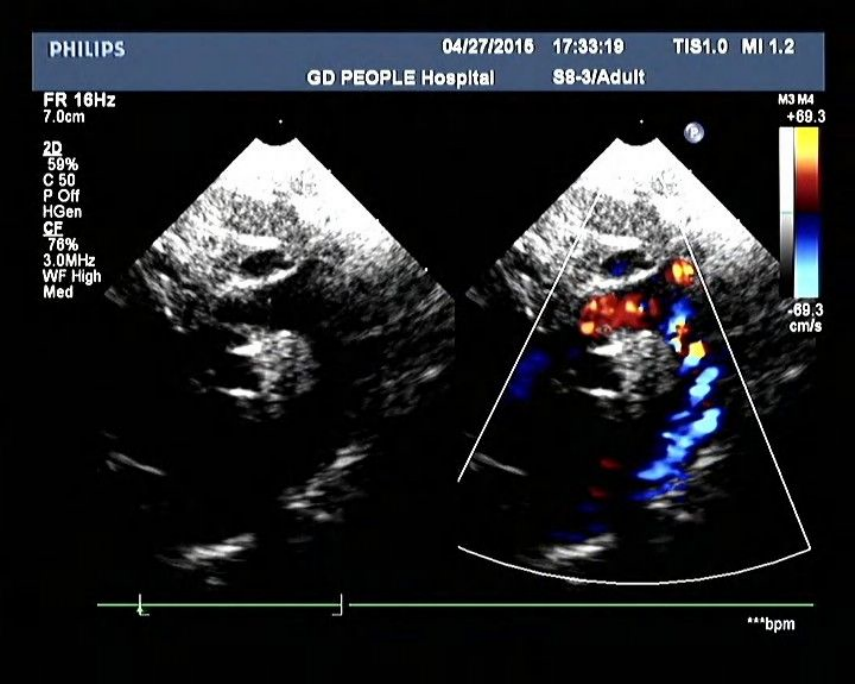

## Slide 20
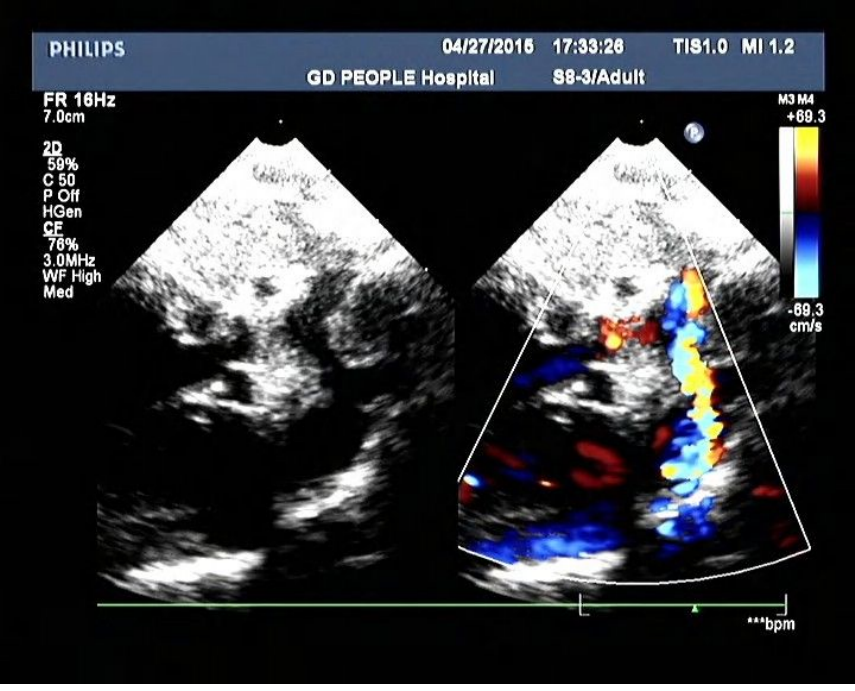

## Slide 21
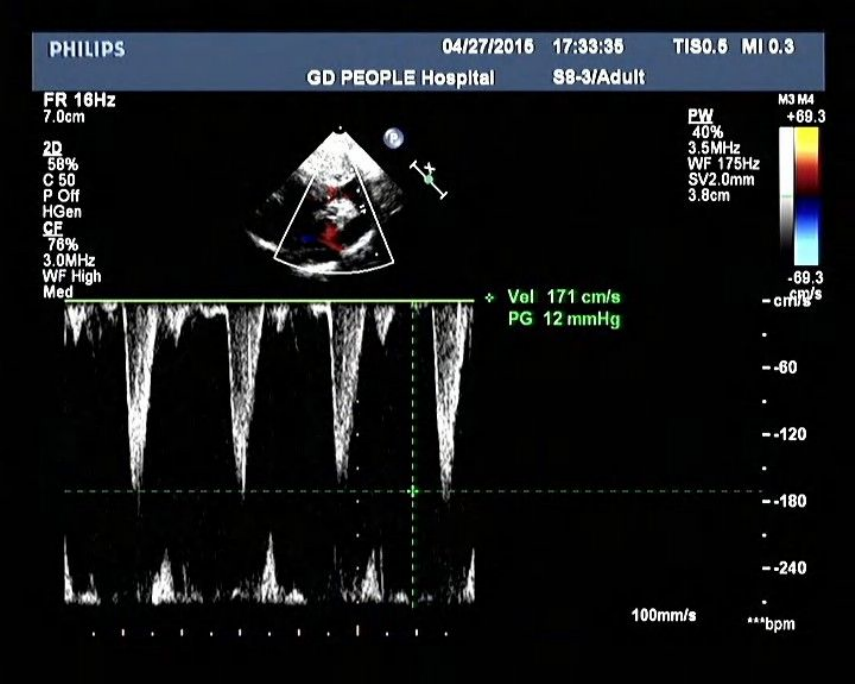

## Slide 22
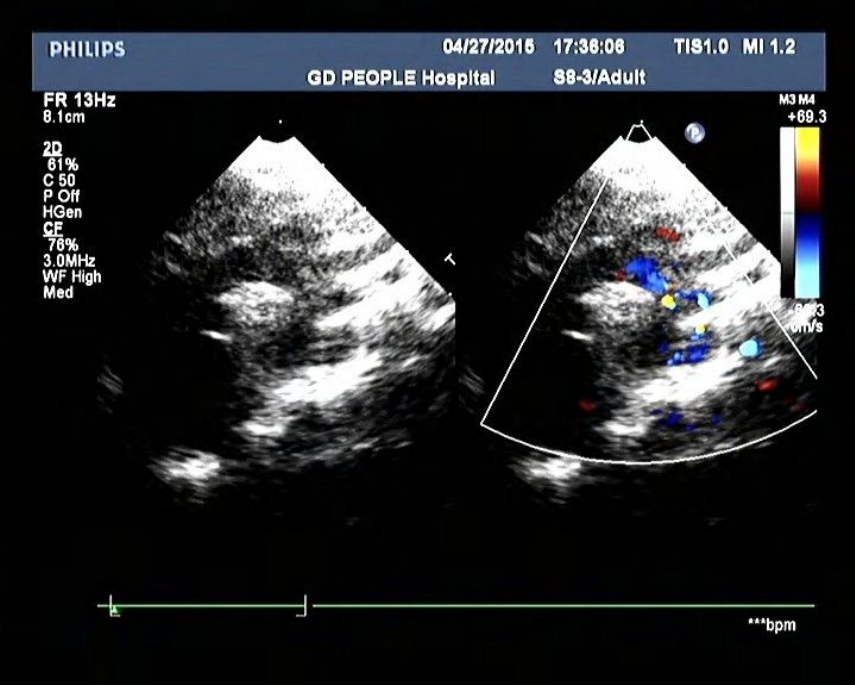

## Slide 23
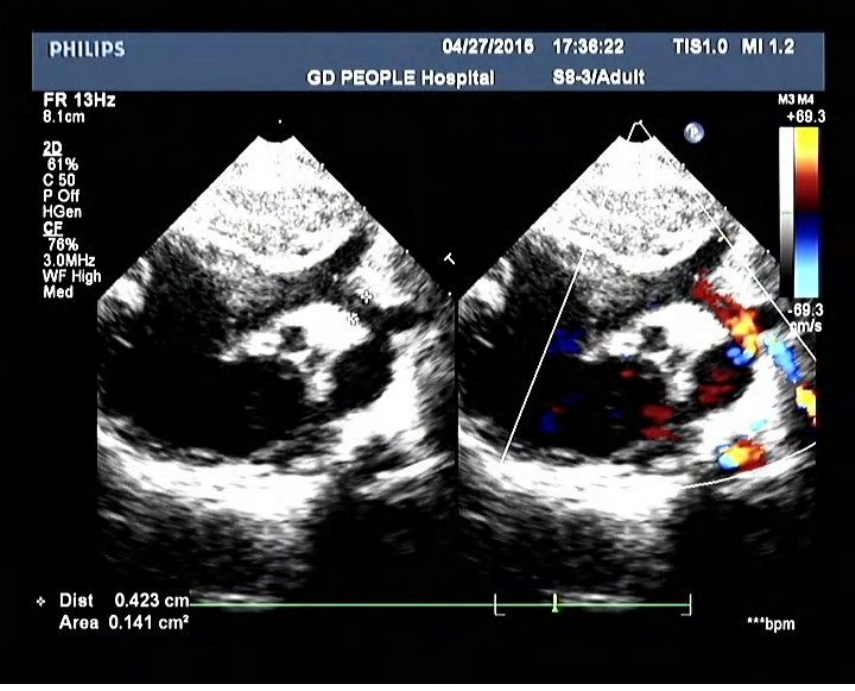

## Slide 24
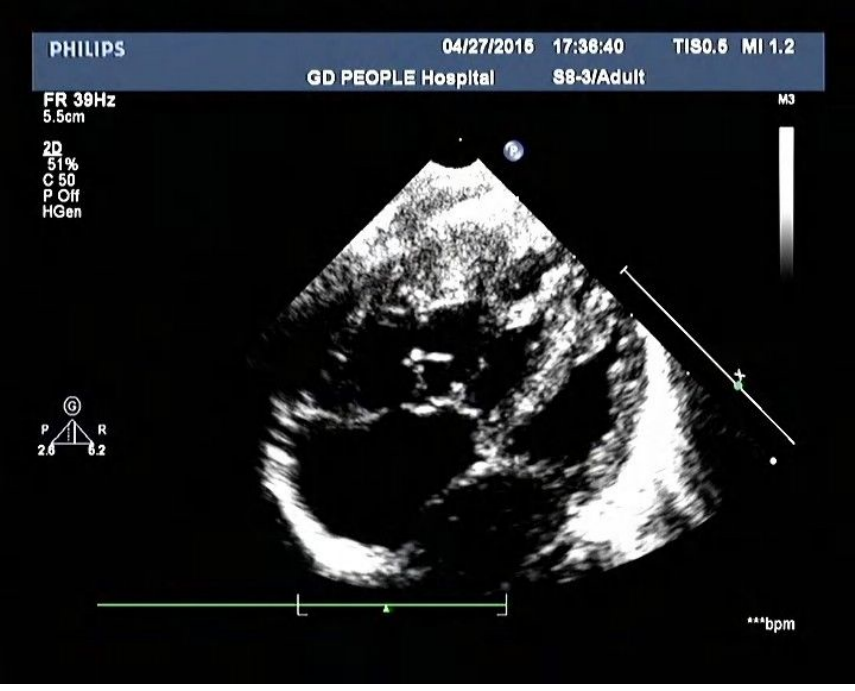

Supplement: Supplementary file 3 — Additional file 1. Online only materials [file 13023_2020_1378_MOESM1_ESM.pptx]
