# Supplementary figures and images for: Role of the ADCY9 gene in cardiac abnormalities of the Rubinstein-Taybi syndrome
Source: Orphanet J Rare Dis. 2020 Apr 22;15:101. doi: 10.1186/s13023-020-01378-9 (PMC7178576; doi:10.1186/s13023-020-01378-9)

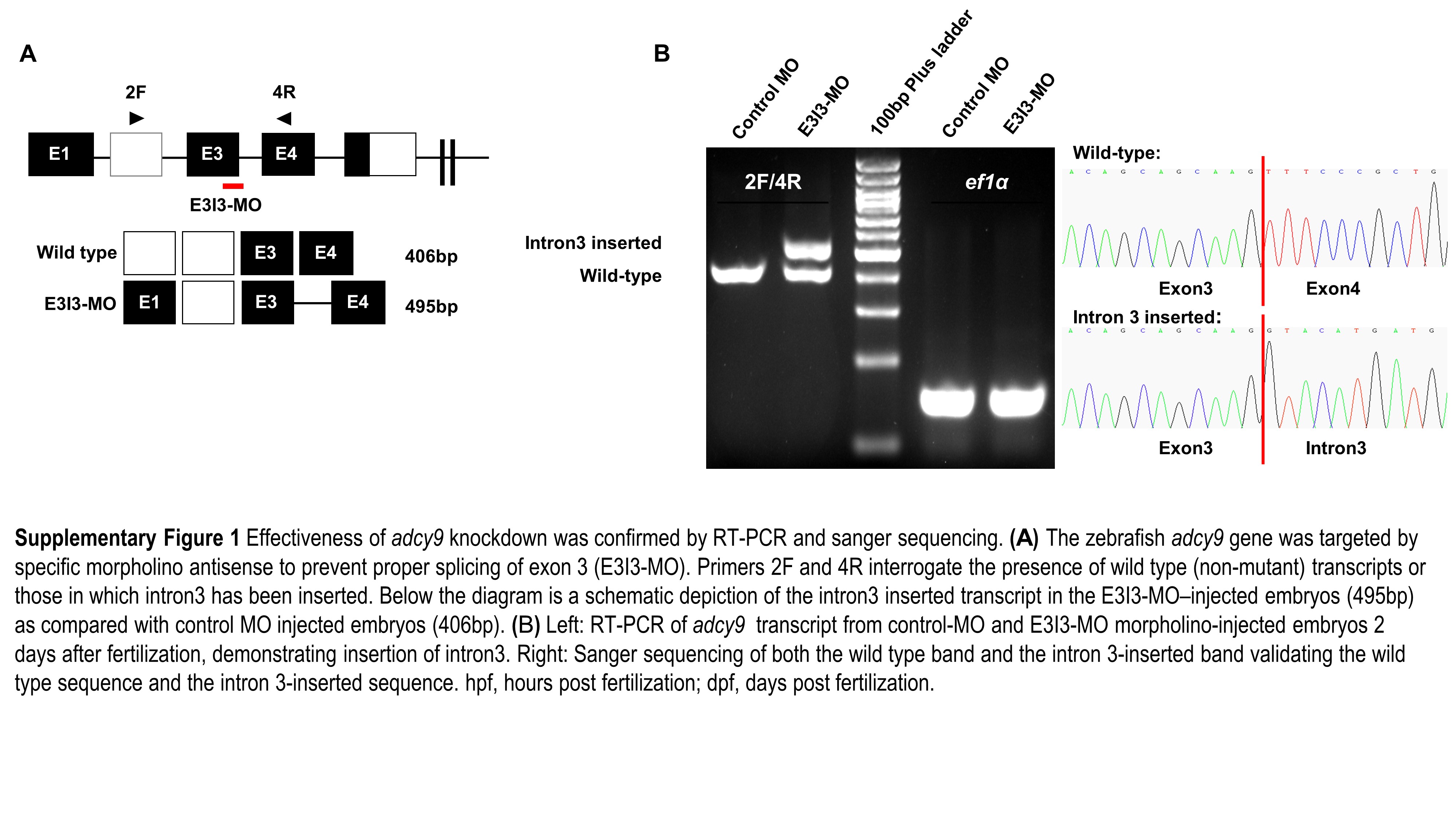

Supplement: Supplementary file 4 — Additional file 2 Supplementary Figure 1. Effectiveness of adcy9 knockdown was confirmed by RT-PCR and sanger sequencing. [file 13023_2020_1378_MOESM2_ESM.jpg]
